# Malignant Transformed and Non-Transformed Oral Leukoplakias Are Metabolically Different

**DOI:** 10.3390/ijms26051802

**Published:** 2025-02-20

**Authors:** Roberta Rayra Martins-Chaves, Victor Coutinho Bastos, Jéssica Gardone Vitório, Filipe Fideles Duarte-Andrade, Thaís dos Santos Fontes Pereira, Flávia Leite-Lima, Thaís Ellen Chaves Gomes, Yuri Abner Rocha Lebron, Victor Rezende Moreira, Monique Sedlmaier França, Lucilaine Valéria de Souza Santos, Liséte Celina Lange, Adriana Nori de Macedo, Carolina Raíssa Costa Picossi, Hélder Antônio Rebelo Pontes, Marina Gonçalves Diniz, Carolina Cavaliéri Gomes, Wagner Henriques de Castro, Gisele André Baptista Canuto, Ricardo Santiago Gomez

**Affiliations:** 1 Graduate Program in Health Sciences, Faculty of Medical Sciences of Minas Gerais (FCMMG), Alameda Ezequiel Dias, 275, Belo Horizonte 30130-110, Minas Gerais, Brazil; falecommonique@hotmail.com; 2 Department of Oral Surgery and Pathology, School of Dentistry, Universidade Federal de Minas Gerais (UFMG), Av. Presidente Antônio Carlos, 6627, Belo Horizonte 31270-901, Minas Gerais, Brazil; coutinhobvictor@gmail.com (V.C.B.); jessicagardone@hotmail.com (J.G.V.); thaissfp@hotmail.com (T.d.S.F.P.); leitelimaflavia@hotmail.com (F.L.-L.); thaisecg@ufmg.br (T.E.C.G.); wagnerhcastro@hotmail.com (W.H.d.C.); 3 Department of Sanitation and Environmental Engineering, School of Engineering, Universidade Federal de Minas Gerais (UFMG), Av. Presidente Antônio Carlos, 6627, Belo Horizonte 31270-901, Minas Gerais, Brazil; yuri_abner@hotmail.com (Y.A.R.L.); victorrznde.eng@gmail.com (V.R.M.); lucilaine@desa.ufmg.br (L.V.d.S.S.); lisete@desa.ufmg.br (L.C.L.); 4 Department of Chemistry, Exact Sciences Institute, Universidade Federal de Minas Gerais (UFMG), Av. Presidente Antônio Carlos, 6627, Belo Horizonte 31270-901, Minas Gerais, Brazil; adrianandm@gmail.com; 5 Chemistry Institute, University of São Paulo, São Paulo 05503-900, São Paulo, Brazil; picossi.carolina@usp.br; 6 Departament of Oral Pathology, João de Barros Barreto University Hospital, Universidade Federal do Pará, Belém 66063-023, Pará, Brazil; harp@ufpa.br; 7 Department of Pathology, Biological Sciences Institute, Universidade Federal de Minas Gerais (UFMG), Av. Presidente Antônio Carlos, 6627, Belo Horizonte 31270-901, Minas Gerais, Brazil; marinadiniz@gmail.com (M.G.D.); carolinacgomes@gmail.com (C.C.G.); 8 Department of Analytical Chemistry, Institute of Chemistry, Universidade Federal da Bahia (UFBA), R. Barão de Jeremoabo, 147, Salvador 40170-115, Bahia, Brazil; gih.canuto@gmail.com

**Keywords:** oral leukoplakia, malignant transformation, oral squamous cell carcinoma, oral carcinogenesis, metabolomics, lipid metabolism, biomarkers

## Abstract

Understanding the early molecular events driving oral carcinogenesis is vital for diagnosing oral squamous cell carcinoma (OSCC) promptly. While metabolic differences between oral leukoplakia (OLK), OSCC, and healthy oral mucosa have been reported, the metabolic changes distinguishing malignant transformed OLKs (MT-OLK) from non-transformed OLKs (NT-OLK) remain unexplored. Here, we examine the metabolomic profiles of 5 cases of MT-OLK and 15 of NT-OLK to identify key predictive molecules using untargeted high-performance liquid chromatography-mass spectrometry. The potentially discriminant compounds were highlighted through a robust statistical analysis workflow, and the dysregulated metabolic pathways were illustrated by enrichment analysis. Seventeen molecular features, primarily lipids—including phospholipids, oxidised lipids, cholesteryl esters, and fatty acids—were identified as discriminants between MT-OLK and NT-OLK across statistical and bioinformatic approaches. Pathway enrichment analysis revealed alterations in lipid metabolism, particularly fatty acid synthesis and degradation, steroid hormone biosynthesis, and glycerophospholipid metabolism. Predictive models showed high accuracy (AUC = 0.88) in distinguishing the two groups. This study suggests that metabolomics has the potential to differentiate between MT-OLK and NT-OLK by identifying candidate biomarkers that may contribute to the understanding of malignant transformation. Validation in larger cohorts is warranted to translate these findings into clinical practice.

## 1. Introduction

Oral leukoplakia (OLK) is the most frequent oral potentially malignant disorder (OPMD); it affects about 4% of the global population [1] and presents a malignisation rate of 9.7% (7.8–11.7) [2,3]. Due to its prevalence and substantial risk of malignant transformation, researchers have extensively studied OLK to understand the mechanisms of oral carcinogenesis and to identify changes that could predict this risk [4,5,6,7,8,9]. Certain molecular alterations, such as chromosomal aberrations and the inactivation of tumor suppressor genes, have been associated with the progression of OLK to oral squamous cell carcinoma (OSCC) [7,10,11]. Nevertheless, the more recognized risk factors for malignant transformation include lesions found in non-smoking females, particularly on the tongue and floor of the mouth, along with a clinically non-homogeneous appearance and high-grade or differentiated oral epithelial dysplasia (OED) [12,13,14,15].

Understanding how OLK transforms into OSCC is vital for prevention strategies. Metabolomics is a promising laboratory approach for identifying biomarkers that can non-invasively identify patients at higher risk for OSCC [16]. It analyzes the products of genomic and proteomic processes, providing a clear picture of a biological system’s phenotype [17]. Given the marked heterogeneity of molecular events during carcinogenesis [18], metabolomics can highlight subtle metabolic shifts throughout the process; and therefore reflect the current state of the analyzed oral mucosa.

Several metabolomic studies have identified molecules potentially linked to the malignant transformation of OLK [19,20,21,22,23,24,25,26,27]. However, all these studies compared OLK with OSCC or samples from healthy individuals. No prior research has used metabolomics to differentiate between malignant transformed (MT) and non-malignant transformed (non-MT) OLK specimens. Distinguishing between MT-OLK and NT-OLK is crucial for understanding the early stages of oral cancer development in the framework of malignant transformation, and has the potential to detect predictive biomarkers associated with this process. Our study compared the metabolomic profile of MT-OLK and NT-OLK, revealing that metabolomics has the potential to differentiate between the two groups whilst also identifying candidate predictive compounds.

## 2. Results

### 2.1. Clinicopathological Characterisation

The study group included 5 samples of MT-OLK and 15 samples of NT-OLK, with a minimum follow-up period of 12 months after the diagnosis. The affected patients (n = 20) were primarily in their third (5/20), fourth (4/20), and seventh (4/20) decades of life (Table 1). The mean age of individuals in the MT-OLK group was approximately 51 years (±17.8), while the mean age for the NT-OLK group was around 56 years (±15.7). Among the MT-OLK cases, three patients were aged 31 to 41, with malignant transformation occurring between 32 and 84 months (average of 40 months, ±22.4). The other two cases involved males in their seventh decade, with a transformation time of 24 months.

Four cases of MT-OLK were found in the tongue (T3, T4, and T5) and the floor of the mouth (T2). Tongue was also the most affected site among the NT-OLK cases (7/15), followed by the buccal mucosa (4), vestibular sulcus (2), hard palate (1), and gingival mucosa (1). Histopathologically, 80% of MT-OLK cases (T2–T5) showed moderate dysplasia, while one case (T1) exhibited severe dysplasia. For NT-OLK cases, 40% had mild dysplasia, 33% had moderate dysplasia, 20% had severe dysplasia, and 7% showed no dysplastic changes. All cases underwent excisional biopsy. Recurrence occurred in 80% of MT-OLK cases (T2–T5) and in about 33% of NT-OLK cases (C1 and C4). One patient (C9) developed a new OLK lesion on the opposite buccal mucosa 72 months after diagnosis. The average time to recurrence for both groups was about 12 months, with follow-up periods ranging from 12 to 144 months (average of 50 months, ±33.72). Detailed clinicopathological information can be found in Table 1.

### 2.2. Data Quality, Processing, and Treatment

The stability of the chromatographic system was assessed by examining the profiles of the quality control (QC) samples and further evaluated through principal component analysis (PCA). The total ion chromatograms of the QC samples exhibited substantial overlap, indicating minimal variation due to instrumental issues. The clustering of QC samples in the PCA plot, observed in both positive and negative ionization modes, suggests that the experiment’s reproducibility remained unaffected (Supplementary Material S1, Figures S1 and S2). Following the optimization of parameters and data preprocessing (Supplementary Material S1, Tables S1 and S2, Figures S3–S6), 5399 molecular features were extracted in positive ionization mode, and 287 molecular features in negative ionization mode. After applying all preprocessing and data pretreatment methods, 1015 molecular features—including 941 detected in positive ionization mode and 74 in negative ionization mode—were evaluated through univariate and multivariate statistical analyses (Supplementary Material S2).

### 2.3. Data Analysis

Of the 1015 molecular features analyzed, 826 exhibited a normal distribution, while 186 showed a non-normal distribution. Hypothesis testing revealed 72 differentially abundant compounds (*p* < 0.05). Among these 72 statistically significant variables, three were more abundant (FC > 2) in the group of transformed OL (M275T1120, M669T1120, and M326T756), while ten were less abundant (FC < −2) (M308T477, M478T701, M476T636, M479T701, M480T704, M501T644, M480T780, M206T275, M478T735, and M782T592) (Supplementary Material S3, Table S4).

**Figure 1:**
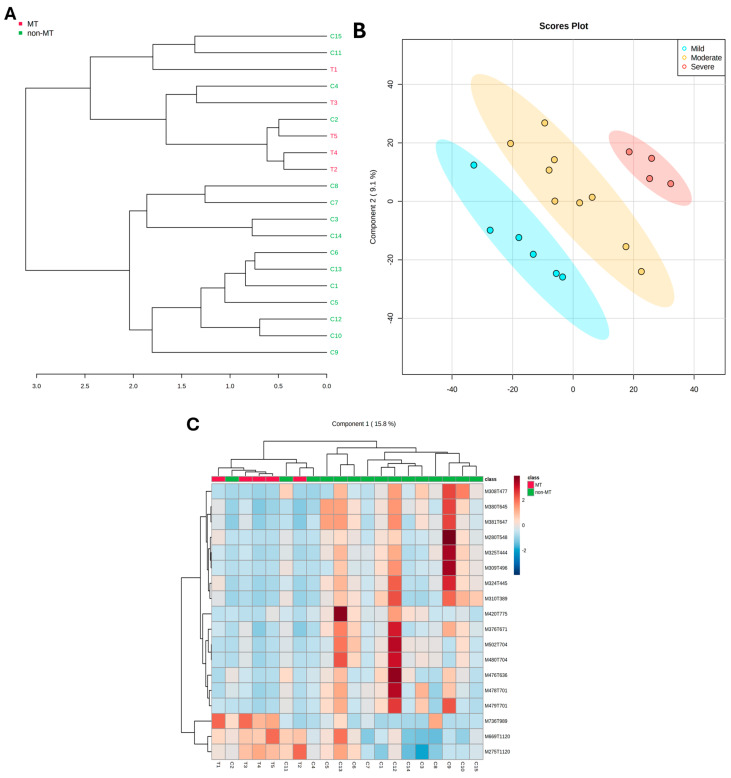
(**A**) Unsupervised hierarchical clustering displayed as a dendogram. Distance measure: Pearson. Clustering algorithm: Mean. MT: malignant transformed. Non-MT: non-malignant transformed. (**B**) 2D scores graph for partial least squares discriminant analysis—PLS-DA. Areas in red, yellow, and blue represent 95% confidence intervals. The graph illustrates clear separation between leukoplakia with varying degrees of dysplasia, with a Q^2^ value of 0.5. (**C**) Unsupervised hierarchical clustering presented as a heatmap shows that non-progressive leukoplakia samples C2, C4, and C11 cluster with transformed leukoplakia samples. However, the groups remain visually distinct overall.

Unsupervised hierarchical clustering of the 72 discriminant compounds (*p* < 0.05) revealed the formation of two distinct clusters. In the dendrogram, the first cluster encompassed all samples of transformed OLK (T1–T5) and 4 out of 15 samples of non-transformed leukoplakias (C2, C4, C11, C15). The remaining non-transformed OL samples (11/15) were grouped in the second cluster (Figure 1A).

While principal component analysis (PCA) and partial least squares discriminant analysis (PLS-DA) did not distinguish the study groups (Supplementary Material S3, Figure S7), when the samples were categorized based on the severity of oral epithelial dysplasia, PLS-DA showed distinct groupings (Q^2^ = 0.5) (Figure 1B). A Significance Analysis of Microarray (SAM) was performed to identify the key compounds responsible for distinguishing between the groups. This analysis revealed 21 molecular features that were significantly different (*p* < 0.05, FDR = 0.2). Of these, 18 were detected in positive ionization mode, while 3 were detected in negative ionization mode (see Supplementary Material S3, Figure S8).

### 2.4. Multivariate Modeling

The MUVR algorithm was employed for minimally biased variable selection in R to further refine discriminant compound identification. This method was essential, as post-test corrections could not be applied to the univariate analyses in this study, and overfitting was observed in the PLS-DA model for group separation (see Supplementary Material S3, Figure S7). In the positive ionization mode, 8 compounds were identified as predictive variables using the random forest (RF) method and 29 using the partial least squares (PLS) method (Supplementary Material S4, Table S6). In negative ionization mode, 5 compounds were classified with RF and 11 with PLS. The permutation tests showed that the models created for variable selection were statistically significant (Supplementary Material S4, Table S8). Eighteen molecular features were consistently identified across univariate analyses, SAM analysis, and MUVR models (Table 2), validating the results and allowing for the selection of these compounds for further identification.

### 2.5. Assessment of Key Molecular Features for Effective Group Discrimination

The 18 selected molecular features were used to create a heatmap. All progressive leukoplakias were clustered together, while samples C2, C4, and C11 were grouped with the transformed leukoplakias, separate from the NT-OLK (Figure 1C). Curiously, sample C2 showed severe OED, and sample C4 had two recurrences, occurring 17 and 20 months after removal (Table 1).

The receiver operating characteristic (ROC) curve was used to evaluate the accuracy of each of the 18 compounds in differentiating between transformed and non-transformed oral leukoplakias. Among these compounds, three were identified as excellent predictors (AUC > 0.9), one was classified as good (AUC > 0.8), six were rated as reasonable (AUC > 0.7), and six were considered weak discriminators (AUC ≥ 0.6). None of the 18 compounds proved ineffective in distinguishing between transformed and non-transformed oral leukoplakias (Table 2).

**Figure 2:**
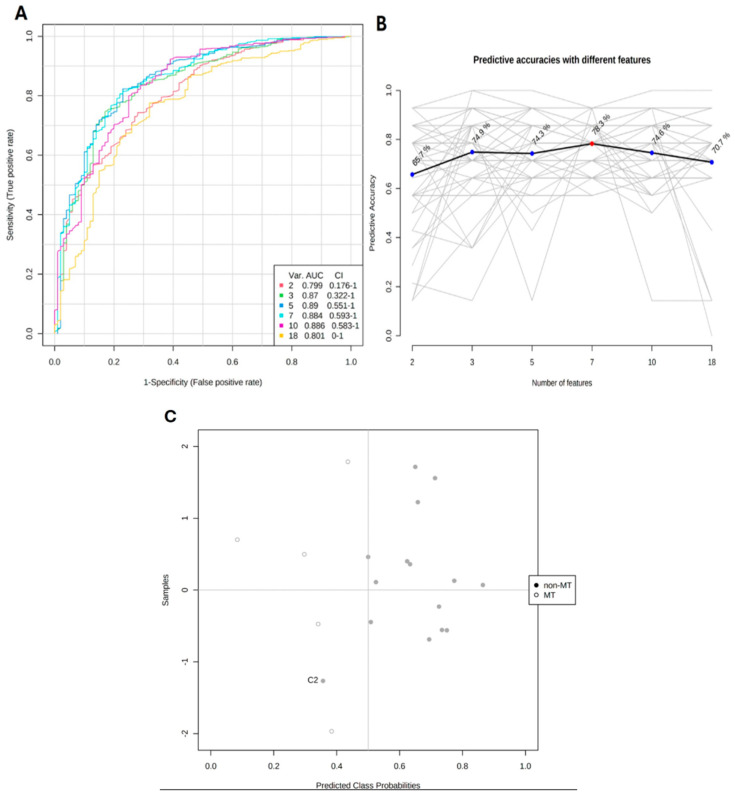
(**A**) ROC curves for the multivariate models built based on average performance from all MCCV (Monte Carlo Cross-Validation) runs. The legend in the bottom right shows the colors of each ROC line, along with the AUC values, confidence intervals (CI), and the number of compounds in each model. (**B**) Predictive accuracy graph of multivariate models. The red dot highlights the most accurate model (Model 4: 7 molecular features; AUC = 0.88; Accuracy 78.3%). (**C**) Scatter plot shows predicted class probabilities from Model 4. The classification threshold is at x = 0.5 due to balanced subsampling. Empty points denote transformed oral leukoplakia samples, while filled points represent non-transformed samples. One non-transformed sample (C2) was misclassified out of 15. Images generated using MetaboAnalyst.

A multivariate algorithm using the Monte Carlo Cross-Validation (MCCV) method was applied to develop a predictive model based on the ROC curve results. The models had predictive values ranging from an AUC of 0.79 to 0.89. The fourth model was the best performer, with an AUC of 0.88 and an accuracy of 78% for predicting the study groups (Figure 2A,B). The model effectively distinguished between transformed and non-transformed oral leukoplakias, as shown in the scatter plot in Figure 2C.

### 2.6. Metabolite Annotation and Pathway Enrichment Analysis

Metabolite annotation was performed using the CEU Mass Mediator tool [28] and the Human Metabolome Database [29]. Annotation information was available for 11 discriminant metabolites as outlined in Supplementary Material S5. The annotated compounds primarily belonged to the lipid class, which includes phospholipids, oxidized lipids, cholesteryl esters, fatty acids, and conjugated classes. Four metabolites—CE(18:2), LysoPE(18:1/0:0), CE(22:5-O), DG(38:1), DG(42:5), and PI(42:6-OH)—have been linked to various cancer types, such as OSCC, breast, stomach, colorectal, prostate, and testicular. However, it is important to note that the identification corresponds to level 3 annotation, according to the Metabolomics Standard Initiative (MSI), with multiple compounds matching some of the features. Therefore, further validation is warranted.

Finally, the mummichog and GSEA algorithms revealed that the enriched metabolic pathways were primarily related to lipid metabolism, particularly in fatty acid synthesis and degradation, C21 steroid hormone biosynthesis and metabolism, squalene and cholesterol biosynthesis, and glycerophospholipid metabolism (Supplementary Material S6, Figure S10, Table S9).

## 3. Discussion

OLK patient management poses significant challenges, from diagnosis through follow-up, due to overlapping characteristics with other oral white plaque lesions and the lack of predictive biomarkers for malignant transformation. Despite the low number of samples, the clinical and histopathological characterization of MT-OLKs revealed some previously described patterns regarding progression and malignancy risk, such as lesion location and dysplasia grade. Four MT-OLKs affected the tongue and one occurred in the floor of the mouth; both high-risk areas for OSCC [30,31,32,33]. Recurrence rates were high, with 80% in MT-OLK compared to 33% of NT-OLKs. Multiple recurrences occurred in 2/5 MT-OLKs, while only 1/15 non-MT case recurred twice. These findings reinforce that recurrence may be a critical predictor of malignant potential, and underscore the importance of thorough post-surgical follow-up.

Younger patients experienced a longer time before malignant transformation compared to older patients, which may align with the neutral and punctuated tumor evolution theories where a prolonged development stage is followed by a rapid onset of genetic aberrations that drive OSCC development [18]. Additionally, a new contralateral lesion in one non-MT case after 72 months of follow-up corroborate the field changes theory, emphasizing the importance of long-term monitoring for OLK [18]. However, a larger cohort with extended follow-up is required to assess these assumptions. Histopathological findings also confirm the existing literature. All MT-OLK showed moderate or severe dysplasia, highlighting the correlation between higher dysplasia grades and increased malignancy risk [12]. However, the presence of moderate or severe dysplasia in 8/15 NT-OLKs endorses oral epithelial dysplasia as just one of several drivers for malignancy [14].

Loss of heterozygosity and inactivation of tumor suppression genes have been the leading events toward oral carcinogenesis [34]. Additionally, considering all the complex layers involved in genotype–phenotype interactions, the last two decades of research have shed light on the metabolic changes as essential mechanisms for OSCC development [35]. While different biomarkers panels based on metabolomic analyses have been proposed for the early diagnosis of high-risk OLKs [20,23,24,25], no definitive list of predictive compounds exists. In the present work, we applied several statistical and bioinformatic methods to find the most refined relevant compounds to distinguish MT-OLKs from non-MT ones.

We found 72 statistically significant molecular features that effectively categorized MT-OLK and NT-OLK groups in hierarchical analysis. Two of the three highly abundant features (FC > 2) in MT-OLKs (M669T1120 and M275T1120) remained significant after multivariate modeling applied for compound filtering and showed the highest accuracy (AUC = 0.96) in distinguishing MT-OLKs from NT-OLKs. These compounds were annotated as Diacylcglycerol—DG(38:1) and N-Dodecylsarcosinate. Although the Human Metabolome Database (HMDB) does not report any physiological effect for these metabolites, diacylglycerol (DAG) is known to regulate tumor initiation, progression, metastasis, and T-cell function, making it important for tumor immunosurveillance [36,37]. Another DAG molecule (DG(42:5)—M736T989) also showed a high predictive value (AUC = 0.95) in our study. Dickinson and colleagues showed that DAG compounds were four times more abundant in OSCC than in healthy controls [38].

N-Dodecylsarcosinate is an N-acylsarcosinate formed by linking sarcosine to a fatty acid chain. Sarcosine compounds were previously related to prostate cancer progression [39,40] and were recently detected in 78–96% of serum samples from prostate cancer patients [41]. Sarcosine successfully differentiates prostate cancer from prostate intraepithelial neoplasia [42] and was recently detected in higher levels in OSCC than in control samples [43]. We believe that DAG and sarcosine compounds may contribute to the malignant transformation of OLK, and further targeted studies are needed to assess their value for predicting OLK risk.

The main goal of metabolomic studies is to find predictive models for the early diagnosis of OSCC. Notably, three non-MT samples were found to cluster with the MT-OLKs in a heatmap based on the 17 discriminant compounds. One sample (C2) was excised from a 76-year-old female pipe smoker and showed severe dysplasia. The other two samples exhibited mild dysplasia, with one (C4) experiencing multiple recurrences. These lesions were monitored for 12 to 36 months and, although no malignant transformation was observed at the time of analysis, they may have a heightened risk of becoming malignant.

The variable selection workflow, which employs comprehensive bioinformatics and statistical methods, is a crucial outcome of this study. We applied Monte Carlo Cross-Validation (MCCV) with the MUVR package to ensure the reliability and generalizability of our predictive models [44]. The MCCV framework allowed us to perform repeated random sampling of the dataset for model training and testing, minimizing biases and overfitting [44,45]. Then, random forest (RF) and partial least squares (PLS) algorithms were applied to identify the discriminant molecular features [44]. In addition to the two previously discussed compounds, we consistently found 16 molecular features across univariate, SAM (Significance Analysis of Microarray) and MUVR (Minimally Unbiased Variable Selection in R) analyses. This consistency using different statistical methods supports the robustness of these molecular features as potential biomarkers.

The prevalence of lipid-related metabolites among the discriminant molecular features corroborated the recently reported critical role of lipids for OLK development and progression [46,47]. A large population-based study revealed that subjects with oral leukoplakia have higher levels of diabetes-related metabolites and an increased LDL/HDL cholesterol ratio [48]. Curiously, five metabolites annotated in our analysis—PI(42:6-OH), PI(42:5-O), DG(38:1), PE(34:1), and 3, 5-Tetradecadiencarnitine—were associated with insulin resistance and diabetes mellitus [49,50,51].

Oxidized phospholipids and specific isoforms of colesteryl ester—CE(18:2), CE(22:5-O), and CE(22:6-OH)—were other important classes of metabolites annotated in our study. Oxidized phospholipids have been associated with a myriad of diseases due to their bioactive effects on numerous cellular pathways [52]. Recent research has linked fatty acid oxidation to chemoresistance in triple-negative breast cancer cells by increasing mitochondrial membrane lipids, which can prevent apoptosis [53]. Moreover, oxidative stress is a primary event for OLK malignant transformation [54,55,56].

Cholesterol accumulation in mitochondria depletes their antioxidant function [57]. Abnormal cholesterol metabolism, reflected by an increase in intracellular cholesteryl esters [58] or altered circulating levels [59,60], is common in malignant neoplasms. Hypocholesterolemia has also been observed in patients with OSCC and several OPMDs [61,62,63]. Kopecka et al. highlighted the role of phospholipids and cholesterol interaction in chemoresistance [64]. Therefore, we propose that there is a synergistic link between lipid metabolism and the increased oxidative stress in MT-OLK cells, which may lead to the development of OSCC.

The predictive models using the discriminant compounds achieved high accuracy, with AUC values ranging from 0.79 to 0.89. These values highlight the effectiveness of the proposed models in distinguishing between MT-OLK and NT-OLK samples. Furthermore, the identified enriched pathways (fatty acid synthesis and degradation, steroid hormone metabolism, and glycerophospholipid metabolism) align with the known metabolic reprogramming observed in cancer; often involving increased lipid biosynthesis to support rapid cell proliferation [35,65,66,67]. These metabolic alterations are likely associated with the malignant transformation of OLK. Although no previous metabolomic study has directly compared MT-OLK and NT-OLK, we have sought to contextualize our findings by drawing parallels with relevant research in the field. The metabolic pathways and compounds identified in our study may serve as a starting point for future investigations exploring potential biomarkers of malignant transformation, contributing to a broader understanding of OLK transformation.

This study has some limitations that must be acknowledged. Despite the rigorous variable selection workflow, we were only able to achieve the putative level 3 metabolite annotation. Therefore, further validation using advanced techniques such as tandem mass spectrometry (MS/MS) is needed. Our sample size is limited; the inclusion of only five MT-OLK samples may not capture the full biological variability of malignant transformation. However, the low frequency of this event inherently limits the availability of such cases. A recent systematic review and meta-analysis reported a pooled malignant transformation rate for OLK of 6.64% worldwide, with an even lower estimated rate of 3.97% for Latin America [68]. These findings highlight the challenge of assembling larger cohorts of MT-OLK cases, particularly in regions with lower transformation rates. While a broader sample would enhance the generalizability of our results, our study provides an initial investigation into the metabolic alterations associated with OLK malignant transformation. Moreover, three NT-OLK samples were followed up for 12 months. Since our study’s mean time for malignant transformation was approximately 45 months, we cannot assume that these NT-OLKs would not undergo malignant transformation. Finally, the retrospective nature of our data retrieval limited the availability of data on lifestyle factors—including tobacco, alcohol use, and diet—preventing their inclusion in the analysis.

Future studies should focus on a targeted longitudinal validation of our findings in larger cohorts to explore the clinical value of the putatively identified compounds to predict OLK transformation.

## 4. Materials and Methods

### 4.1. Ethics and Sample

This cross-sectional study was approved by the Research Ethics Committee of Universidade Federal de Minas Gerais—UFMG (CAAE: 40027620.0.0000.5149) and was carried out under the principles established by the Declaration of Helsinki. A convenience sample of 21 OLK formalin-fixed paraffin-embedded (FFPE) tissue was retrospectively collected. The classification of lesions into NT-OLK (non-transforming oral leukoplakia) or MT-OLK (malignant transforming oral leukoplakia) was determined based on a minimum follow-up period of 12 months. Cases classified as MT-OLK were those in which malignant transformation was confirmed histopathologically. The sample consisted of 6 malignant transformed OLK (MT-OLK) and 15 non-transformed OL (NT-OLK). All included cases were primary OLs and the five MT-OLK underwent malignant transformation into OSCC. Clinical information was collected on age, gender, habits (tobacco and alcohol use), anatomical site, time of evolution, treatment, recurrence, malignant progression, time until malignant progression, and follow-up. Two experienced pathologists (R.S.G) and (R.R.M.C) performed histopathological grading of oral dysplasia. Exclusion criteria were the diagnosis of in situ OSCC. For this reason, one sample of the MT-OLK group was excluded from the study.

### 4.2. Sample Preparation and Metabolite Extraction

The number of sections of each sample was estimated by density calculation to minimize tissue wastage due to the FFPE tissue size variation. The tissue area was measured using Image J software version 1.54 (National Institutes of Health, Bethesda, MD, USA) and used to determine the volume. The number of cuts required was then estimated considering the value of the desired tissue mass per sample (10 mg). We discarded the first two sections of 10 μm to prevent environmental contaminants. Then, 3 to 83 sections of 20 μm were obtained according to the density-based formula predictions. After deparaffinization in four xylene (Merck, Darmstadt, HE, Germany) baths, the samples were centrifuged at 4 °C at 15,000× *g* for 10 min and incubated in a dry bath at 56 °C to dry the xylene. Then, the tissues were re-weighed on an analytical balance to adjust 10 mg of tissue mass per sample. Metabolite extraction was performed using a 200 μL mixture of methanol:chloroform:water (3:1:1, *v*/*v*/*v*) (Sigma-Aldrich, San Luis, MO, USA). Samples were then homogenized for 10 min in an ultrasonic bath, centrifuged at 4 °C at 15,000× *g* for 15 min, and the supernatant was collected and filtered with nylon syringe filters (0.22 μm pore size). Ten microliters from each sample were collected and polled for quality control (QC) preparation. Samples were stored at −80 °C until analysis.

### 4.3. High-Performance Liquid Chromatography and Mass Spectrometry (HPLC-MS)

The HPLC system (LC-20A Prominence, Shimadzu do Brasil, São Paulo, Brazil) was equipped with a C18 reverse phase column (TITAN 18–5 × 2.1 cm id, 1.9 μm, Supelco Discovery HS, Bellefonte, PA, USA) for the separation of the analytes at 40 °C. Ten microliters of each sample were injected into the system in random order. For every four OL samples, 10 µL of the QC sample was injected. For the positive ionization mode (ESI+), we used (A) deionized water (B) acetonitrile, both acidified with 0.1% formic acid, as mobile phases. Deionized water and (B) acetonitrile were used for the negative ionization mode (ESI−). The analysis time was 30 min and the binary gradient occurred as follows: 5 to 95% B for 20 min, followed by 95% B for 3 min, 95% to 20% B for 2 min and 20% B for 5 min for column conditioning [69]. The flow rate was constant at 0.3 mL/min. The chromatography system was coupled to a quadrupole time-of-flight (QTOF) mass analyzer (Bruker MicroTOF QII, Billerica, MA, EUA) using an electrospray ionization source (ESI-micrO QTOF II, Bruker). The detection range of 90 to 1200 Da and an ionization voltage of 4.5 kV (ESI+) and 3.5 kV (ESI−). Nitrogen was used as the desolvation gas at 2 bar and a flow rate of 7.0 L/h. The source temperature was 100 °C and the desolvation temperature was 180 °C. Nitrogen was also used as the drying gas and was generated from the NM32LA nitrogen generator (Peak Scientific, Inchinnan, UK). A full 90–1200 *m*/*z* scan was carried out, using sodium formate as a calibrator.

### 4.4. Data Processing and Treatment

ProteoWizard software (ProteoWizard© version 3, Los Angeles, CA, USA) was used to convert the spectral data acquired into mzXML file. After the chromatogram analysis using Mass++ software Version 3 (Mass Plus Plus, Kyoto, Japan), the pre-processing parameters were defined using the Isotopologue Parameter Optimisation package (IPO—Bioconductor©) package on R software. The optimized parameters were used to process the raw data using the XCMS package (version 3.8.2) in R platform using the centWave method for peak detection, followed by retention time alignment, group and FillPeaks application for missing values imputation (Supplementary Material S1—Table S3) [70]. The data were exported in csv format for treatment. The duplicated molecular features were manually extracted. Molecular features that were missing in more than 20% of the samples in our cohort were excluded, with the remaining missing values being imputed using the k-nearest neighbours (kNN) method by the MetaboAnalyst pipeline (v 5.0). Features with a relative standard deviation greater than 30% in QC samples were also removed and the additional non-parametric RSD filter median/median absolute deviation was applied to remove the remaining noise or non-informative molecular features [71]. Finally, the ESI+ data were normalized by the median and ESI− data by quantile. Log transformation was carried out for both sets. Scaling was not performed for the univariate statistical analyses. For the multivariate statistical analyses, data were normalized by quantile, log-transformed and Pareto scaled.

### 4.5. Statistical Analysis

Univariate statistical analyses were carried out using IBM SPSS Statistics v.26 software. After the Shapiro–Wilk normality test, Welch’s T and Mann–Whitney U tests were applied to compounds with parametric and non-parametric distributions, respectively. T-score and fold-change (FC) were also calculated. *p*-values < 0.05 and FC outside the range of −2 to 2 were deemed significant. Multivariate analyses were performed in MetaboAnalyst v.5.0, in which principal component analysis (PCA) and partial least squares discriminant analysis (PLS-DA) were performed to check instrumental stability and find discriminants, respectively. Supervised and unsupervised hierarchical clustering was applied to find discriminant molecular features. The SAM (Significance Analysis of Microarray) method was used to provide significant molecular features with False Discovery Rate (FDR) control. The multivariate analyses described in this section were carried out on the MetaboAnalyst v4.0 online platform.

### 4.6. Recursive Selection of Discriminating Molecular Features

The MUVR (Minimally Biased Variable Selection in R—from the Swedish University of Agricultural Sciences) [44] algorithm was used for variable selection through a repeated double cross-validation (rdCV) approach. The MUVR package was installed in RStudio (version 1.4.1717), and classification models were built for each dataset using the partial least squares (PLS) and random forest (RF) methods. The results were permuted to determine statistical significance, applying a *p*-value threshold of ≤0.05 over five hundred permutations (nPerm = 500). More details about the modeling parameters and permutation tests, along with the graphs generated, are available in Supplementary Material S4, Tables S5, S7, and S8, Figure S9.

### 4.7. Assessment of the Discriminating Molecular Features Accuracy

The online platform MetaboAnalyst v. 5.0 was used to evaluate the predictive power of the set of molecular features selected in a way that corresponded between the univariate and multivariate statistical methods previously applied. The analysis was conducted using the “Biomarker Analysis” module. The input data consisted of the intensity values of the discriminating molecular features. The ROC (receiver operating characteristics) curve was used to assess the contribution of each compound to the discrimination of the groups under study individually, using the AUC (area under the curve) calculation as a metric. Next, the SVM (support vector machine) multivariate analysis algorithm was used to build models based on the ROC curves previously made. Finally, a scatter plot was constructed to visualize the discriminatory power of the model with the highest AUC value.

### 4.8. Metabolite Annotation

Metabolite annotation was conducted by querying the *m*/*z* values of the 18 discriminant molecular features in publicly available databases using the CEU Mass Mediator tool [28] and the Human Metabolome Database [29]. In positive ionization mode, [M + H], [M + Na], [M + NH_4_], [2M + H], [M + 2H] were included as potential adducts, while in negative ionization mode, we evaluated [M − H], [M + Cl], [M – H + HCOONa], [2M − H], [M − 2H], applying a maximum error threshold of 15 ppm.

### 4.9. Metabolic Pathway Enrichment

The “Functional Analysis” module on the MetaboAnalyst v.5.0 platform was used to enrich metabolic pathways through the integration of mummichog v.2.0 and gene set enrichment analysis (GSEA) algorithms. The input data included *p*-values, mass/charge ratios (*m*/*z*), and t-scores of molecular features identified in both ionization modes. The analyses were performed with a molecular weight tolerance of 10 ppm in mixed analytical mode (positive and negative ionization). Valid *m*/*z* associations were limited to compounds with primary ions, and a *p*-value cutoff of 0.05 was applied. All adducts and isotopes were considered as potential combinations. The analysis utilized the human metabolic flux network (MFN) model, drawing data from KEGG, BiGG, and Edinburgh databases.

## 5. Conclusions

The variable selection by advanced statistical methods enhanced the reliability of the molecular features identified as discriminant compounds. The predictive models developed based on these compounds accurately identified malignant transformed lesions. Further refinement and validation of our findings may help translate these results into practical tools for clinical use, facilitating early detection, risk stratification, and personalized management of OLK.

## Figures and Tables

**Table 1:** Clinical information of the study samples.

#ID	Sex	Age	Ethnicity	Anatomic Location	Evolution (Months)	OED Grade	Malignant Transformation (Months)	Recurrence (Months)	Follow-Up (Months)
T1	F	43	NW	Soft palate and oropharynx	24	Severe	36	No	NI
T2	M	72	W	Floor of the mouth	12	Moderate	24	Yes (2 and 7)	NI
T3	M	73	NW	Tongue (posterior border)	NI	Moderate	NI	Yes (9)	12
T4	F	NI	NI	Tongue (posterior border)	NI	Moderate	NI	NI	NI
T5	F	31	NW	Tongue (posterior border)	2	Moderate	NI	Yes (3, 4, 7, 32)	72
C1	M	54	W	Hard palate	60	Moderate	-	Yes (2 and 2)	48
C2	F	76	NI	Buccal mucosa	NI	Severe	-	NI	NI
C3	M	40	W	Tongue (posterior border)	2	Severe	-	Yes (1)	12
C4	M	73	W	Tongue (posterior border and belly)	NI	Mild	-	NI	NI
C5	M	53	W	Worda antero-posterior de língua	NI	Mild	-	No	24
C6	M	38	W	Tongue (belly)	1	Mild	-	NI	NI
C7	M	69	W	Buccal mucosa	36	Moderate	-	No	72
C8	F	87	W	Fornix	8	Moderate	-	NI	108
C9	F	34	NW	Buccal mucosa	5	Mild	-	Yes (72)	144 *
C10	M	49	W	Tongue (posterior border)	3	Moderate	-	No	60 *
C11	M	69	NW	Buccal mucosa	NI	Mild	-	No	36
C12	M	59	W	Tongue (posterior border and belly)	1	Mild	-	No	24
C13	F	39	NW	Fornix	36	Moderate	-	Yes (6)	84
C14	F	40	NW	Tongue (posterior border and belly)	NI	Severe	-	No	48
C15	M	68	NW	Gingival mucosa	60	Absent	-	No	72

T: group of transformed cases; C: group of control cases; NW: non-white; W: white; NI: not informed. * Lesion regression.

**Table 2:** Molecular features selected based on correspondence criteria from univariate statistical approaches, SAM analysis, ROC curve, and MUVR classification. * Univariate statistical analysis (*p* < 0.05) Significant MUVR modeling (nPerm = 500; *p* < 0.05).

*Molecular Feature*	*p*. Value *	Fold Change	SAM (*p*. Value)	SAM (q. Value)	MUVR (PLS *p*. Value)	MUVR (RF *p*. Value)	AUC	Ionization Mode
M669T1120	0.004	3.2	0.000	0.1	0.08	0.03 *	0.96	Positive
M275T1120	0.003	2.8	0.002	0.4	0.08	0.03 *	0.96	Positive
M736T989	0.005	0.6	0.002	0.4	0.08	0.03 *	0.95	Positive
M376T671	0.007	0.8	0.003	0.4	0.08	-	0.75	Positive
M324T445	0.007	−0.4	0.004	0.4	0.08	-	0.85	Positive
M380T645	0.006	0.7	0.005	0.4	0.08	-	0.6	Positive
M476T636	0.014	−3.5	0.005	0.2	0.003 *	0.002 *	0.68	Negative
M325T444	0.005	0.0	0.007	0.5	0.08	-	0.72	Positive
M308T477	0.003	−2.6	0.007	0.5	0.08	-	0.68	Positive
M478T701	0.013	−3.8	0.007	0.5	0.03 *	0.002 *	0.68	Negative
M310T389	0.009	0.7	0.012	0.5	0.08	-	0.72	Positive
M420T775	0.019	0.4	0.013	0.5	0.08	0.03 *	0.69	Positive
M280T548	0.005	−0.9	0.013	0.5	0.08	-	0.64	Positive
M309T496	0.013	1.0	0.016	0.5	0.08	-	0.72	Positive
M502T704	0.019	0.3	0.016	0.5	0.08	-	0.67	Positive
M480T704	0.021	−3.7	0.016	0.5	0.08	-	0.61	Positive
M479T701	0.015	−2.3	0.025	0.5	0.03 *	0.002 *	0.63	Negative

## Data Availability

The original contributions presented in this study are included in the article/Supplementary Material. Further inquiries can be directed to the corresponding author.

## Supplementary Materials

The following supporting information can be downloaded at: https://www.mdpi.com/article/10.3390/ijms26051802/s1.
