# Supplementary material for: Malignant Transformed and Non-Transformed Oral Leukoplakias Are Metabolically Different
Source: Int J Mol Sci. 2025 Feb 20;26(5):1802. doi: 10.3390/ijms26051802 (PMC11898866; doi:10.3390/ijms26051802)
Supplement: Supplementary file 1 [file ijms-26-01802-s001.zip › SupplementaryMaterial_S1.pdf]

Data quality, processing and treatment

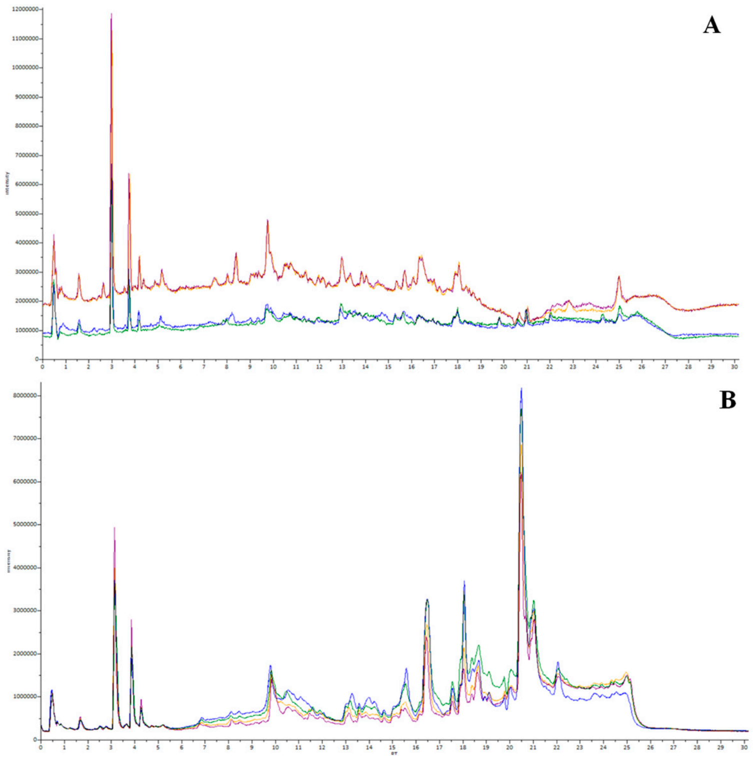

Figure S1. Linearity of the chromatographic run. Total ion chromatograms of QC samples are presented, with the Y-axis showing ion detection intensity and the X-axis indicating retention time. (A) Negative ionization mode. (B) Positive ionization mode. Screenshots taken from Mass ++ software.

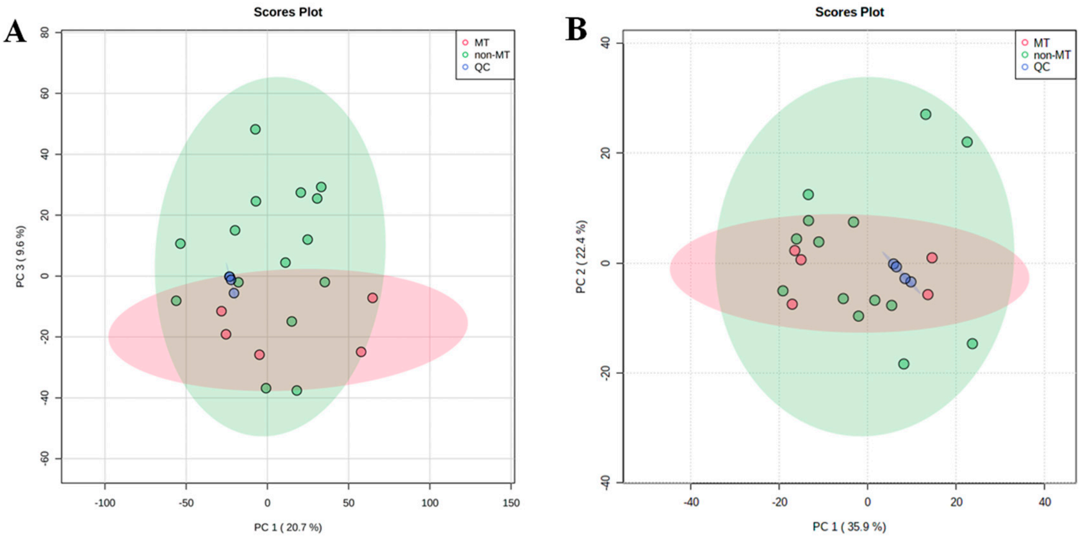

Figure S2. Principal Component Analysis (PCA) shows the grouping of QC samples (blue dots). The variance explained by each component is shown in brackets. Red and green areas mark the 95% confidence interval. (A) Score graph for principal components 3 and 1 in positive ionization mode. (B) Score graph for principal components 2 and 1 in negative ionization mode. PC: principal component; MT: malignant transformed; non-MT: non-malignant transformed; QC: quality control. Images were generated using the MetaboAnalyst platform.

Table S1. Data pre-processing with IPO and XCMS software. Values for optimising the parameters of the data obtained in positive ionisation mode

Optimisation of parameters and data preprocessing

Analysis step	Parameter	Optimisation interval	Set Value
Peak detection	ppm	35-36	35.1
	min_peakwidth	22.5-24	23.25
	max_peakwidth	68-71	71.3
	mzdiff	0.00140-0.00150	0.00152
	snthresh	5.5-6	5.6
	noise	0-80	1
	value_of_prefilter	245-260	8.3
	prefilter	7.5-8.5	260
Correction of retention time and grouping	gapInit	0.8-1.2	0.728
	gapExtend	1.9-2.7	2.7
	profStep	0.7-1	0.754
	bw	0-15	2.25
	minfrac	0.7-0.9	0.89
	mzwid	0.020-0.030	0.026

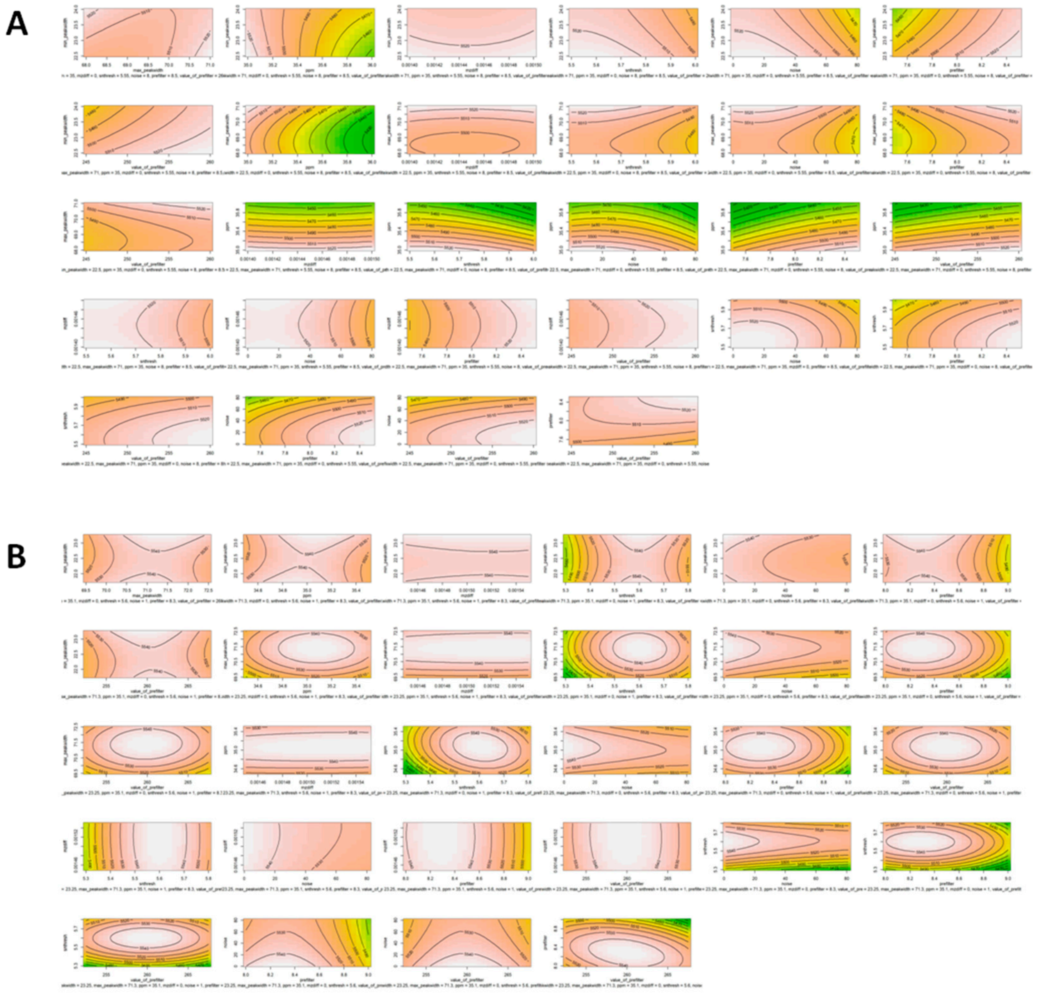

Figure S3. Response surface plots for optimizing peak detection parameters in positive ionisation mode. (A) Plots using standard optimisation intervals from the software. (B) Plots using intervals defined in the first optimisation. The light pink regions of the graph indicate suitable parameter values.

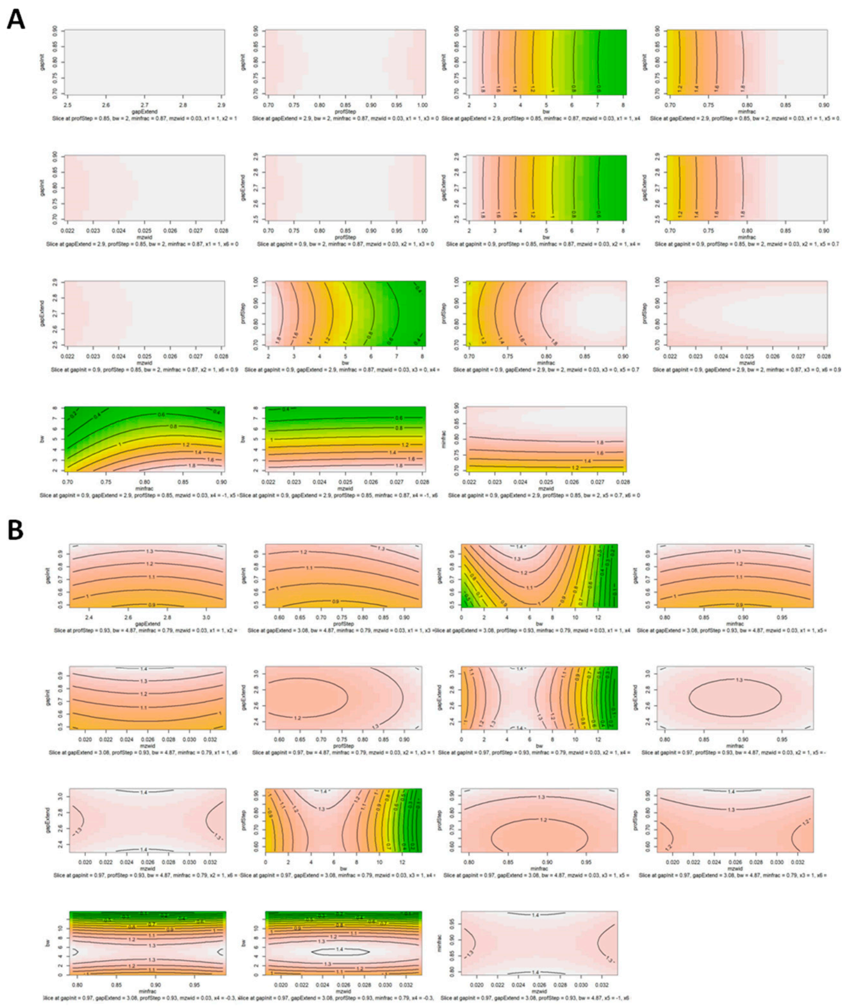

Figure S4. Response surface plots for optimising retention time correction parameters in positive ionisation mode. (A) Plots using standard optimisation intervals from the software. (B) Plots using intervals defined in the first optimisation. The light pink regions of the graph indicate suitable parameter values.

Table S2. Data pre-processing with IPO and XCMS software. Values for optimising the parameters of the data obtained in negative ionisation mode

Analysis step	Parameter	Optimisation interval	Set Value
Peak detection	ppm	49-52	52.3
	min_peakwidth	18-20	21
	max_peakwidth	123-127	128.2
	mzdiff	0.0052-0.0056	0.0054
	snthresh	08-10*	9
	noise	250-300	292
	value_of_prefilter	0-150	2.5
	prefilter	1-4*	75
Correction of retention time and grouping	gapInit	0.3-0.4	0.4
	gapExtend	1.8-2.4	2.1
	profStep	0.55-0.85	0.85
	bw	40-50	40
	minfrac	0.8-0.9	0.85

	mzwid	0.025-0.040	0.025

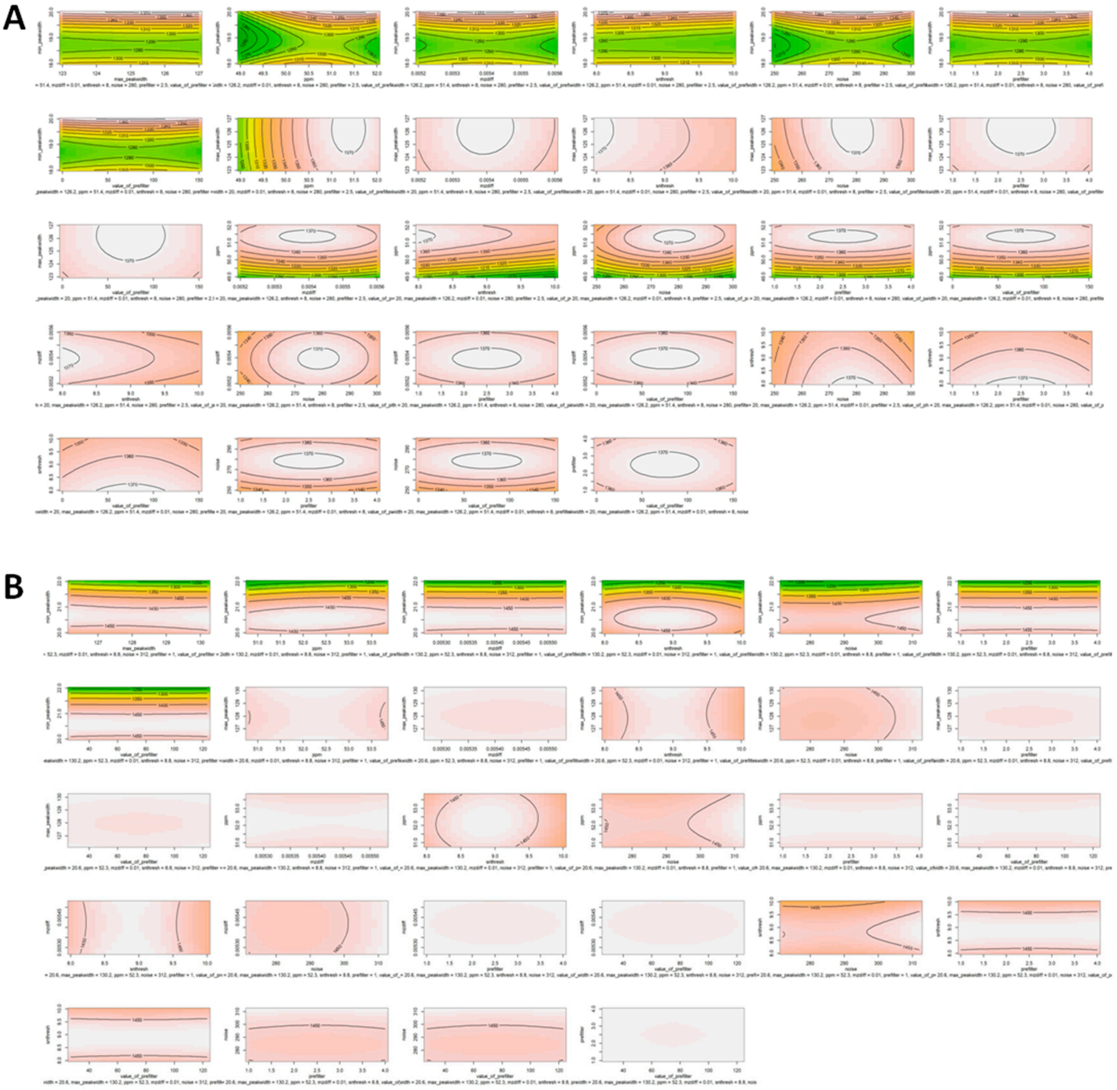

Figure S5. Response surface plots for optimising peak detection parameters in negative ionisation mode. (A) Plots using standard optimisation intervals from the software. (B) Plots using intervals defined in the first optimisation. The light pink regions of the graph indicate suitable parameter values.

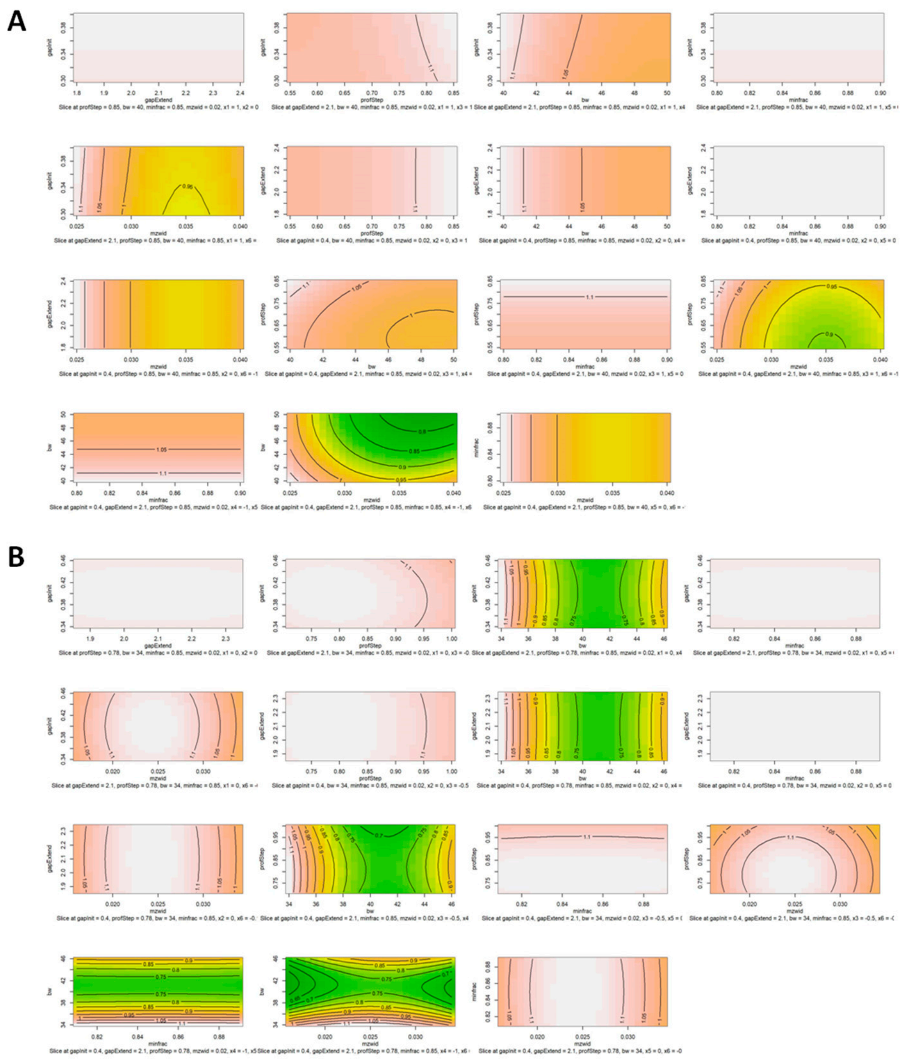

Figure S6. Response surface plots for optimising retention time correction parameters in negative ionisation mode. (A) Plots using standard optimisation intervals from the software. (B) Plots using intervals defined in the first optimisation. The light pink regions of the graph indicate suitable parameter values.

Table S3: Command lines used for data pre-processing in the XCMS software.

Step	Positive ionisation mode	Negative ionisation mode
Package call	library(xcms)	library(xcms)
Peak detection	xsetpos1 <- xcmsSet(method="centWave",peakwidth=c(23.25,71.3),ppm=35.1,noise=1,snthresh=5.6,mzdiff=0.00152,prefilter = c(8.3,260),mzCenterFun="wMean",integrate=1,fitgauss = FALSE,verbose.columns = FALSE)	xsetneg1 <- xcmsSet(method="centWave",peakwidth=c(21,128.2),ppm=52.3,noise=292,snthresh=9,mzdiff=0.0054,prefilter = c(2.5,75),mzCenterFun="wMean",integrate=1,fitgauss = FALSE,verbose.columns = FALSE)
Peak grouping	xsetpos2<- group(xsetpos1,method="density",bw=2.25,mzwid=0.026,minfrac=0.7,minsamp=1,max =50)	xsetneg2<- group(xsetneg1,method="density",bw=40,mzwid=0.025,minfrac=0.85,minsamp=1,max =50)
Retention time correction	xsetpos3 <- retcor(xsetpos2,method="obiwarp",plottype="deviation",distFunc="cor_opt",profStep=0.754,center=22,response=1,gapInit= 0.728,gapExtend= 2.7,factorDiag= 2,factorGap= 1,localAlignment=0)	xsetneg3<- retcor(xsetneg2,method="obiwarp",plottype="deviation",distFunc="cor_opt",profStep=0.85,center=23,response=1,gapInit=0.4,gapExtend= 2.1,factorDiag= 2,factorGap= 1,localAlignment=0)
Peak grouping	xsetpos4<- group(xsetpos3,method="density",bw=2.25,mzwid=0.026,minfrac=0.7,minsamp=1,max =50)	xsetneg4<- group(xsetneg3,method="density",bw=40,mzwid=0.025,minfrac=0.85,minsamp=1,max =50)
Noise exclusion	xsetpos5 <- fillPeaks(xsetpos4)	xsetneg5 <- fillPeaks(xsetneg4)
Statistical analysis and table construction	reporttab<- diffreport(xsetpos5,”casos”,”controles”,”qcs_2”,100)	reporttab<- diffreport(xsetneg5,”casos”,”controles”,”qcs”,100)
