# Supplementary material for: Malignant Transformed and Non-Transformed Oral Leukoplakias Are Metabolically Different
Source: Int J Mol Sci. 2025 Feb 20;26(5):1802. doi: 10.3390/ijms26051802 (PMC11898866; doi:10.3390/ijms26051802)
Supplement: Supplementary file 1 [file ijms-26-01802-s001.zip › SupplementaryMaterial_S3.pdf]

**Table S4.** Differentially abundant compounds classified by *p*-values in ascending order. *m/z: mass/charge ratio. **R.T: retention time. §: differentially abundant compounds according to the fold change calculation.

| **Ionisation mode** | **Molecular feature** | ***p*. value** | ***m/z**** | ***R.T.**** | **t.score** | **fold change** |
|---|---|---|---|---|---|---|
| Positive | M310T1120 | 0.002 | 310.3085 | 1119.5500 | 17.6269 | 0.9975 |
| Positive | M311T1120 | 0.002 | 311.3100 | 1119.5500 | 9.6889 | 0.7482 |
| Positive | M308T477 | 0.003 | 308.2998 | 477.2765 | 0.2443 | **-2.6412**§ |
| Positive | M275T1120 | 0.003 | 275.2658 | 1119.5550 | 5.7776 | **2.8427**§ |
| Positive | M669T1120 | 0.004 | 668.6128 | 1119.5500 | 4.6028 | **3.1981**§ |
| Positive | M325T444 | 0.005 | 325.2991 | 444.0415 | 1.1243 | -0.0317 |
| Positive | M736T989 | 0.005 | 735.5581 | 988.9240 | 6.2511 | 0.6254 |
| Positive | M280T548 | 0.005 | 280.2590 | 547.9390 | 0.2256 | -0.9180 |
| Positive | M380T645 | 0.006 | 380.3617 | 645.2285 | 10.7906 | 0.6564 |
| Positive | M293T1120 | 0.006 | 293.2782 | 1119.5400 | 1.2883 | 0.4734 |
| Positive | M324T445 | 0.007 | 324.2988 | 444.8560 | 3.2590 | -0.3550 |
| Positive | M482T783 | 0.007 | 482.3237 | 783.1750 | 2.2905 | -0.6257 |
| Positive | M376T671 | 0.007 | 376.3650 | 671.1120 | 4.9782 | 0.8245 |
| Positive | M310T389 | 0.009 | 310.2831 | 388.7310 | 1.9445 | 0.6923 |
| Positive | M266T287 | 0.010 | 266.2543 | 287.2615 | 1.2443 | 1.4254 |
| Positive | M210T265 | 0.011 | 210.1813 | 264.6640 | 1.2399 | -0.0433 |
| Negative | M478T701 | 0.013 | 478.3126 | 700.5520 | 33.4965 | **-3.7919**§ |
| Positive | M309T496 | 0.013 | 309.2839 | 496.3915 | 0.8746 | 1.0234 |
| Negative | M476T636 | 0.014 | 476.2885 | 635.6680 | 29.0667 | **-3.5066**§ |
| Positive | M345T218_2 | 0.015 | 345.2616 | 218.2370 | 5.3014 | -0.0925 |
| Negative | M479T701 | 0.015 | 479.3137 | 700.8190 | 53.7392 | **-2.3204**§ |
| Positive | M326T756 | 0.017 | 326.3750 | 755.7100 | 4.1682 | **2.2087**§ |
| Positive | M369T695 | 0.018 | 369.1826 | 694.7750 | 3.3534 | 0.3862 |
| Positive | M296T305 | 0.019 | 296.2665 | 305.4040 | 1.5042 | 0.2051 |
| Positive | M420T775 | 0.019 | 420.3906 | 774.9380 | 5.1578 | 0.3628 |
| Positive | M502T704 | 0.019 | 502.2898 | 704.4380 | 3.0951 | 0.3084 |
| Positive | M393T631 | 0.020 | 393.3643 | 631.4805 | 10.4944 | 1.7841 |
| Positive | M409T789 | 0.020 | 409.3920 | 788.8010 | 3.5241 | 0.0040 |
| Positive | M480T704 | 0.021 | 480.3064 | 703.6470 | 3.8792 | **-3.7299**§ |
| Positive | M350T635 | 0.022 | 350.3509 | 634.9625 | 8.2003 | 1.6220 |
| Positive | M503T644 | 0.023 | 503.2942 | 643.5050 | 3.0356 | 0.9974 |
| Positive | M364T691 | 0.024 | 364.3638 | 690.7100 | 2.1680 | 0.6860 |
| Positive | M610T254 | 0.024 | 610.4616 | 253.8030 | 1.2310 | 0.5331 |
| Positive | M524T740 | 0.025 | 524.2988 | 740.4405 | 3.3033 | -1.6675 |
| Positive | M282T265 | 0.025 | 282.2520 | 264.5460 | 2.7073 | 0.6929 |
| Positive | M387T209_1 | 0.025 | 386.5084 | 209.3025 | 5.8376 | 1.0153 |
| Positive | M381T647 | 0.026 | 381.3632 | 646.6430 | 7.3122 | 0.6845 |
| Positive | M460T218_1 | 0.026 | 459.6833 | 218.1180 | 19.9925 | 0.8639 |
| Negative | M501T644 | 0.027 | 501.3004 | 643.6890 | 33.0222 | **-2.4458**§ |
| Positive | M309T152_2 | 0.027 | 308.9015 | 152.4020 | 0.9669 | -0.0757 |
| Positive | M502T644 | 0.028 | 502.2917 | 643.5500 | 4.8635 | 0.4163 |
| Positive | M351T636 | 0.029 | 351.3508 | 636.0410 | 4.6472 | 1.3802 |

| | | | | | | |
|---|---|---|---|---|---|---|
| Positive | M690T218 | 0.030 | 689.5207 | 218.0730 | 6.2525 | -0.5128 |
| Positive | M577T195 | 0.030 | 576.9360 | 194.8930 | 6.9245 | -1.2395 |
| Positive | M418T665 | 0.031 | 418.3759 | 664.9250 | 2.1324 | -0.8136 |
| Positive | M492T652 | 0.032 | 492.3066 | 651.5110 | 1.9702 | -0.4296 |
| Positive | M396T736 | 0.032 | 396.3782 | 736.3315 | 5.6475 | 0.7416 |
| Positive | M259T456 | 0.033 | 259.1477 | 455.9705 | 0.6856 | 0.9075 |
| Positive | M311T389 | 0.034 | 311.2827 | 388.5540 | 0.8737 | -0.1490 |
| Positive | M392T195_1 | 0.034 | 391.6209 | 194.8925 | 5.5446 | 1.4890 |
| Positive | M526T834 | 0.035 | 526.3136 | 834.2330 | 3.0977 | -0.2602 |
| Positive | M576T195_1 | 0.035 | 575.9350 | 194.8930 | 14.8336 | -0.1413 |
| Positive | M339T497 | 0.035 | 339.3171 | 496.6480 | 1.7990 | 1.1244 |
| Negative | M480T780 | 0.036 | 480.3277 | 780.0165 | 34.3858 | **-2.4821**§ |
| Positive | M781T592 | 0.037 | 780.5482 | 592.2190 | 4.4063 | -0.0800 |
| Positive | M609T235 | 0.038 | 609.4634 | 235.0100 | 8.9823 | 1.3298 |
| Positive | M467T218_1 | 0.039 | 466.6757 | 218.0730 | 0.7063 | 0.4978 |
| Positive | M392T631 | 0.039 | 392.3615 | 631.1130 | 13.5795 | -1.4551 |
| Positive | M388T624 | 0.040 | 388.3270 | 624.3700 | 3.6514 | -0.3547 |
| Positive | M381T837 | 0.042 | 381.2140 | 836.6940 | 1.1463 | 0.3809 |
| Positive | M700T217 | 0.043 | 699.5070 | 217.3770 | 1.5953 | -0.2661 |
| Positive | M253T265 | 0.043 | 253.2226 | 264.5345 | 0.2982 | 0.4298 |
| Positive | M206T275 | 0.044 | 206.1877 | 275.3175 | 0.3928 | **-2.4370**§ |
| Positive | M568T740 | 0.045 | 568.2678 | 739.6785 | 0.8473 | 0.4830 |
| Positive | M478T735 | 0.046 | 478.3197 | 734.8950 | 3.7517 | **-2.1069**§ |
| Positive | M494T722 | 0.046 | 494.3220 | 722.4015 | 2.8542 | 1.4696 |
| Positive | M782T592 | 0.047 | 781.5506 | 592.2220 | 3.9697 | **-3.7269**§ |
| Positive | M562T805 | 0.047 | 562.3285 | 804.6325 | 0.4824 | 1.2105 |
| Positive | M689T217 | 0.048 | 689.0179 | 217.4750 | 0.0635 | 0.0996 |
| Positive | M500T636 | 0.048 | 500.2723 | 635.9920 | 0.0804 | 0.3373 |
| Positive | M481T704 | 0.049 | 481.3066 | 703.6315 | 0.4941 | 1.0075 |
| Positive | M689T218 | 0.049 | 688.5173 | 217.6900 | 1.6277 | 0.7860 |

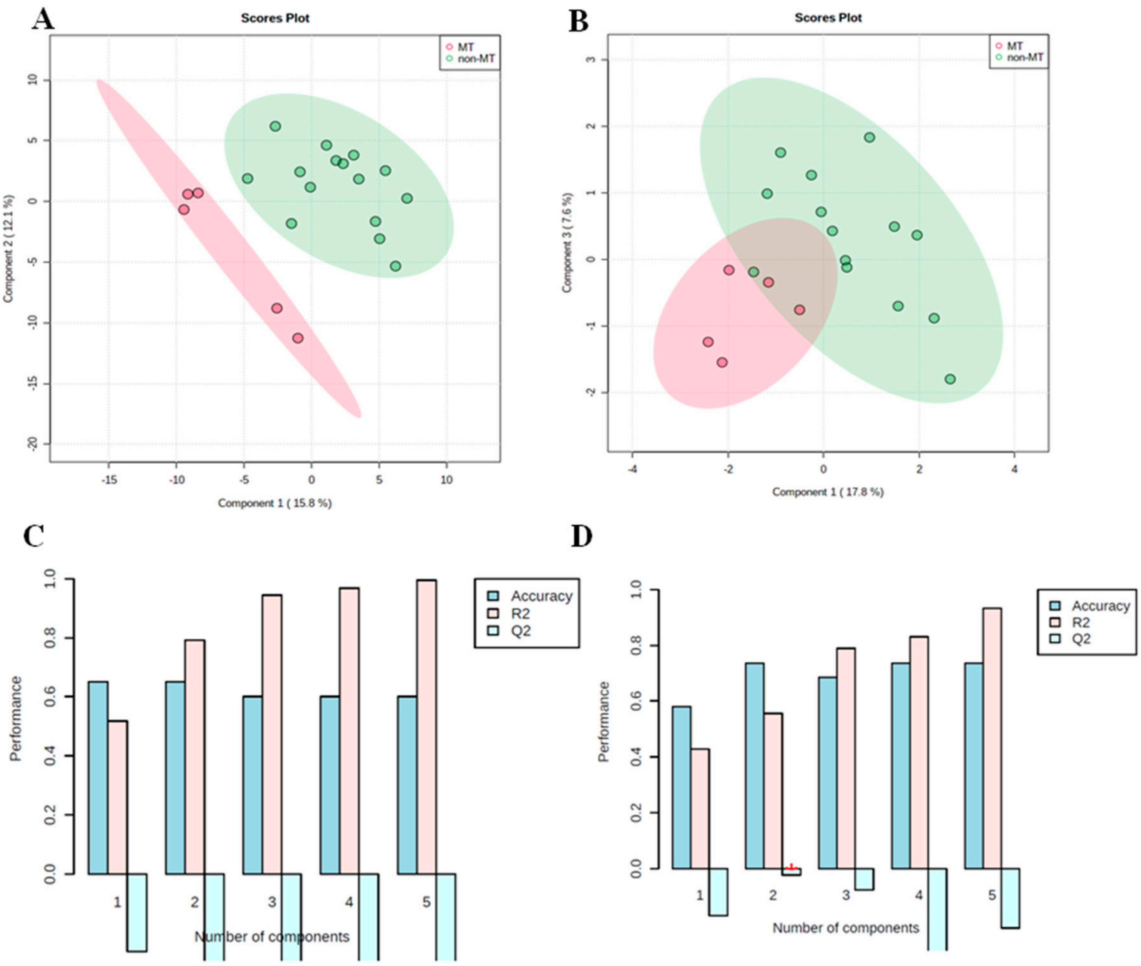


**Figure S7.** Partial least squares discriminant analysis - PLS-DA using data from positive (A) and negative (B) ionisation modes. The explained variance for each component is indicated in brackets. Red dots represent transformed leukoplakia samples, while green dots indicate non-transformed samples, with the respective areas showing 95% confidence. The graphs demonstrate clear separation between MT and non-MT OLKs. Histograms (C) and (D) show Q2 values for each component as light blue bars. Both models exhibited negative Q2 values, indicating potential overfitting. The image was generated using the MetaboAnalyst platform.

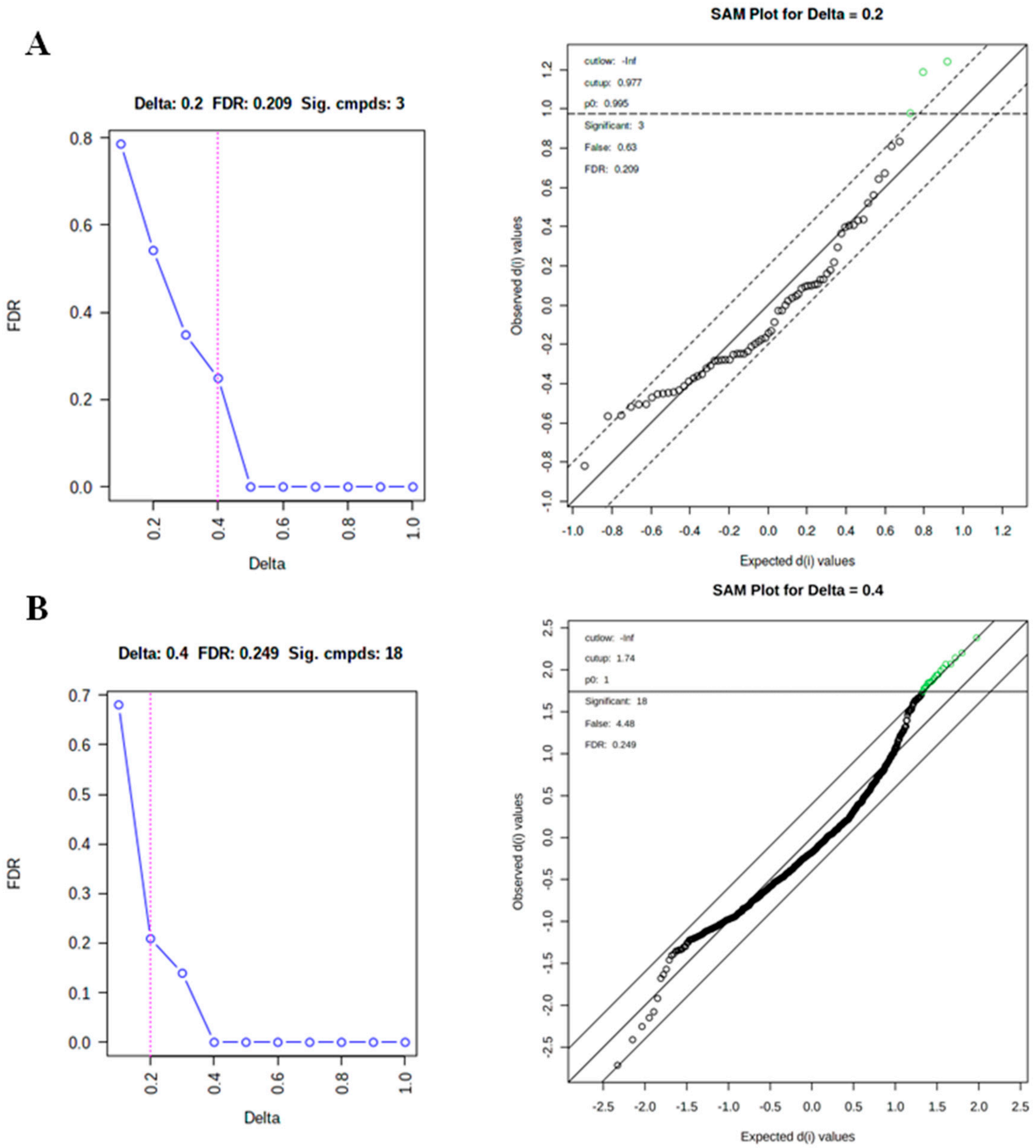


**Figure S8.** The SAM analysis is shown with graphs on the left that demonstrate control of false discoveries by adjusting the Delta value. The scatter plots on the right display the observed relative difference (y-axis) versus the expected relative difference from permuted data (x-axis). The central diagonal line represents equality between these measurements, while the outer lines indicate Delta distance from the center. The green dots represent the discriminating compounds. (A) Data in negative ionisation mode: three significant compounds. (B) Data in positive ionisation mode: eighteen significant compounds. Screenshots are from MetaboAnalyst.
