# Supplementary material for: Malignant Transformed and Non-Transformed Oral Leukoplakias Are Metabolically Different
Source: Int J Mol Sci. 2025 Feb 20;26(5):1802. doi: 10.3390/ijms26051802 (PMC11898866; doi:10.3390/ijms26051802)
Supplement: Supplementary file 1 [file ijms-26-01802-s001.zip › SupplementaryMaterial_S4.pdf]

Table S5. Command lines for <u>variable classification modeling</u> of data obtained in positive and negative ionisation mode.
Multivariate modelling – Minimmally Biased Variable Selection in R (MUVR)

Input data	Common commands	PLS	RF
Positive	filePath <- file.choose(input_muvr_pos) input_muvr_pos<- read_excel(filePath) y <- input_muvr_pos [,1][[1]] x <- input_muvr_pos [,-1] library(doParallel) library(MUVR) nCore <- detectCores()-1 cl<- makeCluster(nCore) registerDoParallel(cl) stopCluster(cl)	muvrModel <- MUVR(X = x, Y = y, nOuter = 5, nInner = 4, nRep = 90, varRatio = 0.8, method = 'PLS', fitness= 'BER', scale= 'TRUE')	muvrModel <- MUVR(X = x, Y = y, nOuter = 5, nInner = 4, nRep = 100, varRatio = 0.8, method = 'RF', fitness= 'BER', scale= 'TRUE')
Negative	filePath <- file.choose(input_muvr_neg) input_muvr_neg<- read_excel(filePath) y <- input_muvr_neg [,1][[1]] x <- input_muvr_neg [,-1] library(doParallel) library(MUVR) nCore <- detectCores()-1 cl<- makeCluster(nCore) registerDoParallel(cl) stopCluster(cl)	muvrModel <- MUVR(X = x, Y = y, nOuter = 5, nInner = 4, nRep = 80, varRatio = 0.85, method = 'PLS', fitness= 'BER', scale= 'TRUE')	muvrModel <- MUVR(X = x, Y = y, nOuter = 5, nInner = 4, nRep = 90, varRatio = 0.8, method = 'RF', fitness= 'BER', scale= 'TRUE')

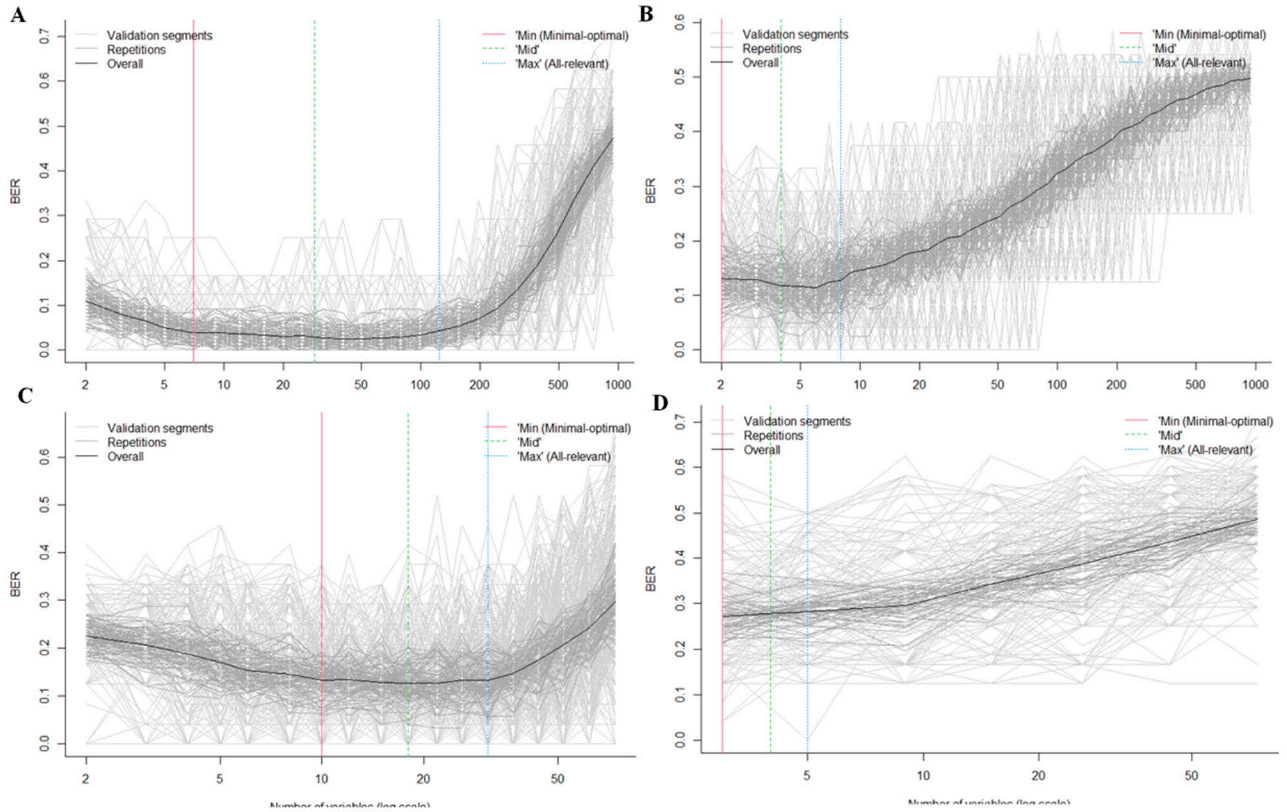

Figure S9. Validation plots for MUVR classification modeling for data obtained in positive (A and B) and negative (C and D) ionisation mode. The light gray lines represent the validation performance for the individual inner segments, while the dark gray lines represent the inner segment validation curves averaged over the repetitions. The red, green and blue dotted lines represent the minimum, average and maximum number of variables selected, respectively. The model error rate (BER) is represented by the Y-axis. The Y-axis represents the number of variables included for internal modeling. A and C. PLS method. B and D. RF method.

Table S6. Predicting variables selected for data obtained in positive and negative ionisation mode. RF: Random Forest. PLS: Partial Least Squares. (+): Positive ionisation mode. (-): Negative ionisation mode.

**Classification method**	**Molecular feature (+)**	**Ranking**	**Molecular feature (-)**	**Ranking**
RF	m669t1120	1	m478t701	1
	m275t1120	2	m479t701	2
	m539t235	3	m476t636	3
	m311t1120	4	m423t563	4
	m609t235	5	m501t644	5
	m736t989	6	-	-
	m478t636	7	-	-
	m420t775	8	-	-
PLS	m669t1120	1	m476t636	1
	m310t1120	2	m478t701	2
	m311t1120	3	m479t701	3
	m275t1120	4	m501t644	4
	m308t477	5	m480t780	5
	m736t989	6	m1187t780	6
	m325t444	7	m1189t780	7
	m293t1120	8	m492t719	8
	m539t235	9	m615t780	9
	m482t783	10	m432t564	10
	m266t287	11	m558t802	11
	m376t671	12	-	-
	m380t645	13	-	-
	m478t636	14	-	-
	m420t775	15	-	-
	m280t548	16	-	-
	m502t704	17	-	-
	m792t1078	18	-	-
	m309t496	19	-	-
	m369t695	20	-	-
	m503t644	21	-	-
	m539t234	22	-	-
	m324t445	23	-	-
	m191t818	24	-	-
	m310t389	25	-	-
	m838t192	26	-	-
	m210t265	27	-	-
	m524t740	28	-	-
	m480t704	29	-	-

Table S7. Command lines for permutation testing of the models generated to classify the variables of data obtained in positive and negative ionisation modes.

Input data	PLS	RF
Positive	actual=MUVR(X = x, Y = y, nOuter = 5, nInner = 4, nRep = 90, varRatio = 0.8, method= 'PLS', fitness= 'BER', scale= 'TRUE')<br><br>actua1Fit=actual$miss[model]<br><br>for (p in 1:nPerm) {<br>cat('\n Permutation',p,'of',nPerm)<br>YPerm=sample(y)<br>perm= MUVR(X = x, Y = y, nOuter = 5, nInner = 4, nRep = 90, varRatio = 0.8, method= 'PLS', fitness= 'BER', scale= 'TRUE')	actual=MUVR(X = x, Y = y, nOuter = 5, nInner = 4, nRep = 80, varRatio = 0.8, method= 'RF', fitness= 'BER', scale= 'TRUE')<br><br>actua1Fit=actual$miss[model]<br><br>for (p in 1:nPerm) {<br>cat('\n Permutation',p,'of',nPerm)<br>YPerm=sample(y)<br>perm= MUVR (X = x, Y = y, nOuter = 5, nInner = 4, nRep = 80, varRatio = 0.8, method= 'RF', fitness= 'BER', scale= 'TRUE')
Negative	actual=MUVR(X = x, Y = y, nOuter = 5, nInner = 4, nRep = 80, varRatio = 0.85, method= 'PLS', fitness= 'BER', scale= 'TRUE')<br><br>actua1Fit=actual$miss[model]<br><br>for (p in 1:nPerm) {<br>cat('\n Permutation',p,'of',nPerm)<br>YPerm=sample(y)<br>perm= MUVR(X = x, Y = y, nOuter = 5, nInner = 4, nRep = 80, varRatio = 0.85, method= 'PLS', fitness= 'BER', scale= 'TRUE')	actual=MUVR(X = x, Y = y, nOuter = 5, nInner = 4, nRep = 90, varRatio = 0.8, method= 'RF', fitness= 'BER', scale= 'TRUE')<br><br>actua1Fit=actual$miss[model]<br><br>for (p in 1:nPerm) {<br>cat('\n Permutation',p,'of',nPerm)<br>YPerm=sample(y)<br>perm= MUVR(X = x, Y = y, nOuter = 5, nInner = 4, nRep = 90, varRatio = 0.8, method= 'RF', fitness= 'BER', scale= 'TRUE')

Table S8. Results of the permutation tests for the models generated to classify the variables of data obtained in positive and negative ionisation modes.

Input	PLS	RF
Positive	0.08	0.03*
Negative	0.003*	0.002*
