# Supplementary material for: Malignant Transformed and Non-Transformed Oral Leukoplakias Are Metabolically Different
Source: Int J Mol Sci. 2025 Feb 20;26(5):1802. doi: 10.3390/ijms26051802 (PMC11898866; doi:10.3390/ijms26051802)
Supplement: Supplementary file 1 [file ijms-26-01802-s001.zip › SupplementaryMaterial_S6.pdf]

Pathway enrichment analysis

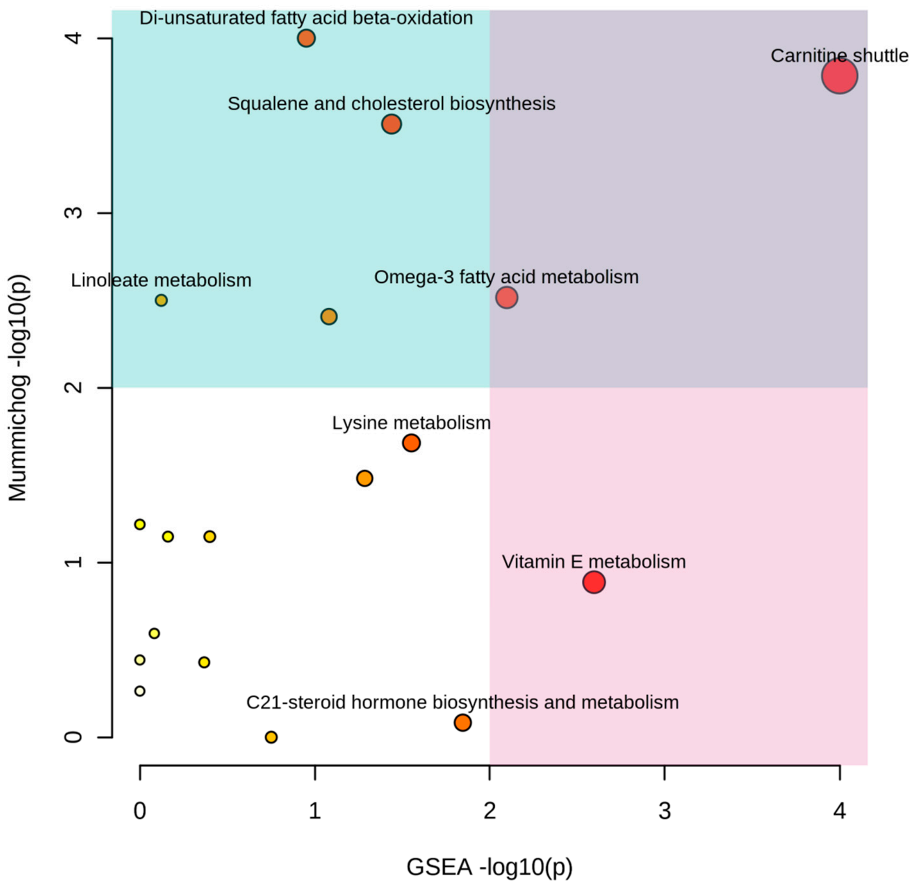

Figure S10. The graphs from the MetaboAnalyst Functional Analysis module show the results of the Fisher method for combining p-values from mummichog (Y-axis) and GSEA (X-axis). Circle size and color reflect their combined p-values: large red circles represent the most disturbed pathways. Blue and pink areas indicate pathways significant to mummichog and GSEA, respectively, while the purple area highlights pathways significant to both. Mass accuracy is 10 ppm.

Table S9. Metabolic pathways enriched in the combined analysis by the mummichog and GSEA algorithms. Total Size: number of compounds in the pathway. Hits: Number of constituent compounds of the pathway possibly present in the leukoplakia samples analyzed. Sig. Hits: number of compounds from the pathway potentially present in the leukoplakia samples analyzed. Mummichog *p.* values: values from the analysis using the mummichog algorithm. GSEA *p.* values: values from the analysis using the GSEA algorithm. Combined *p.* values: values adjusted to the study data by combining the two algorithms in a Gamma distribution.

**Enriched pathways**	**Total Size**	**Hits**	**Sig. Hits**	**Mummichog *p.* values**	**GSEA *p.* values**	**Combined *p.* values**
Carnitine shuttle	72	25	3	0.2866	0.0099	0.0195
Vitamin E metabolism	54	15	1	0.6935	0.0500	0.1512
Omega-3 fatty acid metabolism	39	7	1	0.4220	0.0889	0.1607
Squalene and cholesterol biosynthesis	55	15	2	0.3118	0.1900	0.2266
Di-unsaturated fatty acid beta-oxidation	26	4	1	0.2684	0.3333	0.3054
Lysine metabolism	52	10	1	0.5439	0.1667	0.3083
C21-steroid hormone biosynthesis and metabolism	112	47	2	0.8862	0.1188	0.3423
Fatty acid activation	74	32	3	0.4363	0.2871	0.3855
Glycosphingolipid metabolism	67	11	1	0.5786	0.2268	0.3978
Linoleate metabolism	46	19	2	0.4241	0.8673	0.7357
Vitamin A (retinol) metabolism	67	30	1	0.9089	0.4200	0.7494
Fatty Acid Metabolism	63	13	1	0.6405	0.6300	0.7697
Bile acid biosynthesis	82	38	2	0.7980	0.6535	0.8610
De novo fatty acid biosynthesis	106	13	1	0.6405	0.8300	0.8675
Prostaglandin formation from arachidonate	78	42	3	0.6270	0.9990	0.9194
Glycerophospholipid metabolism	156	18	1	0.7589	0.9082	0.9458
Aspartate and asparagine metabolism	114	20	1	0.7947	0.9990	0.9771
Leukotriene metabolism	92	23	1	0.8389	0.9990	0.9861
